# Return to homeland: Bereaved caregiver perceptions of immigrant patient quality of death after repatriation to their country of origin, a retrospective cohort study

**DOI:** 10.1017/S1478951526102612

**Published:** 2026-06-05

**Authors:** Ana I. Flores, Frances Eichholz Heller, Andreea I. Dinicu, Fiona Angel, June Hou, Paul Maciejewski, Holly Prigerson, Craig David Blinderman, Ana I. Tergas

**Affiliations:** 1 Palliative Care, Department of Medicine, Penn State College of Medicine, Hershey, PA, USA; 2 Palliative Care, Department of Medicine, Columbia University Irvine Medical Center, New York, NY, USA; 3 Obstetrics and Gynecology Institute, Cleveland Clinic, Cleveland, OH, USA; 4 Columbia University Vagelos College of Physicians and Surgeons, New York, NY, USA; 5 Division of Gynecologic Oncology, Department of Obstetrics and Gynecology, Columbia University Irving Medical Center, New York, NY, USA; 6 Cornell Center for Research on End-of-Life Care, Weill Cornell Medicine, New York, NY, USA; 7 Department of Radiology, Weill Cornell Medicine, New York, NY, USA; 8 Department of Medicine, Weill Cornell Medicine, New York, NY, USA; 9 Division of Supportive and Acute Care Services, Memorial Sloan Kettering, New York, NY, USA; 10 Division of Gynecologic Oncology, Department of Obstetrics and Gynecology, Rutgers New Jersey Medical School, Newark, NJ, USA

**Keywords:** Immigrant health, repatriation, end-of-life care, quality of death, Latinx/Latino patients, caregiver perceptions

## Abstract

**Objectives:**

Terminally ill immigrant patients may wish to return to their homeland to die. However, the challenges and repatriation experiences faced by terminally ill Latinx immigrants who return to their country of origin (COO) remain underexplored. This study examines bereaved caregiver accounts of end-of-life (EOL) care and repatriation experiences of Latinx immigrants who returned to their COO following a terminal diagnosis.

**Methods:**

Bereaved caregivers were interviewed via telephone. Primary outcomes were patient repatriation prior to death and caregiver perceptions of patient quality of death (QOD). Secondary outcomes included repatriation barriers, symptom burden and management, health-care utilization, and location of death. Caregivers of deceased patients treated at a U.S. urban tertiary medical center from 2013 to 2020 who requested and returned to their COO for death were included. Deceased patient clinical and sociodemographic information was obtained from the chart review.

**Results:**

Nineteen caregivers of patients who returned to their COO were included, most (*n* = 15/19, 78.9%) from the Dominican Republic. Two patients experienced logistical challenges during travel to their COO. None received supplementary nutrition or ventilatory support. Most patients had well-controlled symptoms (12/19 had pain, 11/12 pain was controlled) and died peacefully with dignity (84.2%) at their preferred location (73.7%). Most caregivers reported that their loved one was happy with repatriation (89.5%) and that repatriation improved EOL quality of life (84.2%). QOD was rated favorably (average score of 4.2/5).

**Significance of results:**

QOD was favorable after repatriation. Successful repatriation is feasible and an important component of QOD that should be included in goals of care discussions.

## Introduction

The Hispanic/Latinx population is the fastest growing minority group in the United States (U.S.) (Passel and Cohn 2022). This demographic faces disproportionately poor end-of-life (EOL) care outcomes (Voelker 2012; Siegel et al. 2021; Passel and Cohn 2022). Compared to non-Hispanic whites, Latinx immigrants are less likely to receive EOL care in accordance with their preferences and are more likely to receive aggressive EOL care that does not lead to improved quality of life (Taylor et al. 2017). Quality of death (QOD) is broadly defined as a concept encompassing symptom control, avoidance of non–beneficial aggressive treatments, alignment of care with patient preferences, and psychosocial and spiritual well–being in the final days of life (Zhang et al. 2012; Curtis et al. 2013), Latinx immigrants are also more likely to have a poor QOD compared to non-Hispanic whites in the U.S. (Silva et al. 2016; Yarnell et al. 2017; Shen et al. 2019; Tergas et al. 2022a). Therefore, studies focused on integration of EOL preferences into care plans and optimization of EOL care are needed for Latinx immigrants.

Despite the desire of many immigrants to return to their countries of origin (COO) at the EOL (Pan et al. 2004; Selsky et al. 2012; Aisporna and Erickson-Hurt 2019), there are a limited number of studies reporting on the challenges faced by Latinx immigrants who attempt to repatriate to their COO after receiving a terminal diagnosis (indicating EOL), and most of those studies are limited to case reports (Jaramillo and Hui 2016; Karapetyan et al. 2018; Metchnikoff et al. 2018; Tergas et al. 2022a; Kawai et al. 2024). Prior case reports of Latinx and other immigrant patients seeking repatriation highlight recurring challenges, including limited insurance/financial resources, difficulties obtaining medical clearance for commercial travel, fear of deportation or inability to return to the U.S., insufficient social support for travel coordination, and communication gaps with clinicians regarding feasibility. Collectively, these reports suggest repatriation is often strongly value–driven yet hindered by structural, legal, and logistical barriers. Given that Latinx immigrants are less likely to receive EOL care in accordance with their preferences and are more likely to have a reduced QOD (Silva et al. 2016; Yarnell et al. 2017; Shen et al. 2019; Tergas et al. 2022a), there is a need to better understand and support EOL preferences for Latinx immigrants to improve their EOL quality of life and death, particularly those who wish to return to their COO at EOL.

Given the paucity of literature on repatriation before EOL and the significant impact that repatriation may have on patients who wish to repatriate after a terminal diagnosis, a more complete understanding of the EOL needs of Latinx immigrants is warranted. By interviewing caregivers of deceased patients, we examined and characterized the repatriation and EOL experiences of Latino immigrants who expressed interest in returning to their COO after a terminal diagnosis by retrospective chart review and caregiver telephone interview. We hypothesized that Latinx immigrants who were able to return to their COO at the EOL may have had meaningful or positive EOL experiences, as perceived by their bereaved caregivers.

## Methods

### Sample

The study cohort was comprised of bereaved caregivers of patients with a diagnosis of a terminal illness who were seen by the palliative care inpatient consult service between 2013 and 2020 at an academic medical center in New York City (NYC), and who expressed a desire to return to their COO for their death to the palliative care team. The caregivers of these patients were contacted by telephone in January and February 2022 and asked to participate in a telephone-based interview. Patients with caregivers who declined study participation were excluded. Chart review and caregiver interviews indicated that the desire to return to the COO was an explicit, value–based patient preference. No cases involved deportation orders, involuntary return, or externally imposed pressures (e.g., insurance termination or forced cost–based decisions).

### Data collection and outcome measures

Data collection and outcome measures were obtained by retrospective chart review from the electronic medical record and telephone interview with the bereaved caregivers. The patients’ electronic medical records were reviewed to obtain patient date of birth, sex, COO, U.S. residency status, insurance status, race, ethnicity, religion, partnership status, number of children, primary terminal illness diagnosis, stage of cancer if applicable, and patient comorbidities. The caregiver surveys were conducted via telephone in the caregiver’s primary language (English or Spanish), by bilingual members of the research team (AF, FE). A total of three attempts were made to contact the caregivers.

The telephone-based structured interview of the caregivers took approximately 30–45 min to complete. The primary outcomes were patient repatriation prior to death and caregiver perceptions of patient satisfaction regarding their decision to repatriate at EOL and their experiences with repatriation. Secondary outcomes included: barriers to transportation from the U.S. to the COO or within the COO, factors contributing to inability to return to COO, satisfaction with decision of returning or not returning to COO, other barriers to returning to COO, location of death (hospital, ICU, hospice, home), symptom burden and management, health-care utilization at EOL, pursuit of alternative treatments, time to death after arrival in COO, and overall patient QOD (rated on a scale of 1–5, where 1 means poor and 5 means best possible) in the last week of life. Descriptive statistics were used to summarize the collected data.

### Research ethics and patient consent

The protocol was approved by Columbia University Irving Medical Center IRB AAAT6113. All procedures were performed in accordance with the ethical standards of the institution, national research committee, and the 1964 Declaration of Helsinki and International Conference on Harmonization Guidelines for Good Clinical Practice and later amendments. Verbal informed consent was obtained from all participants of this study.

## Results

### Patient and caregiver dyads

**Figure 1.**
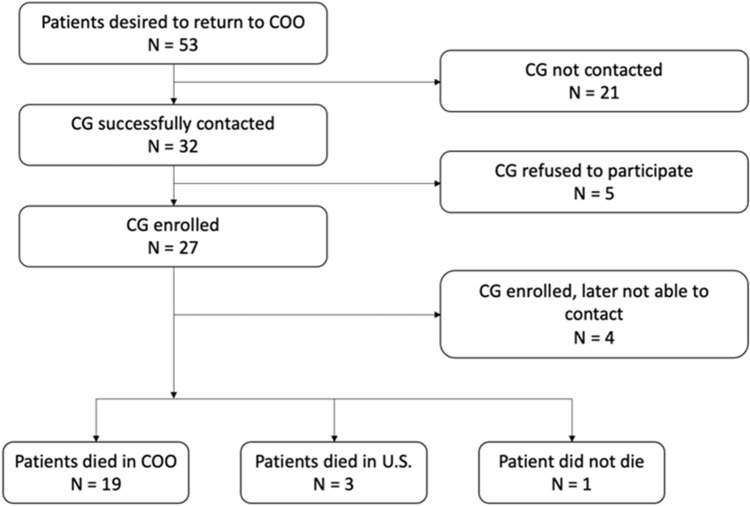
Flowchart showing patient and caregiver enrollment and outcomes for returning to COO. Figure 1 long description.

Fifty-three (53) eligible patients with a terminally ill diagnosis who were seen by palliative care service and expressed a desire to return to their COO were identified by retrospective chart review. The study team successfully contacted 32 of those patients’ caregivers, and 27 (84.4%) consented to the study. However, 4 caregivers were not able to be reached to complete the interview after they had initially consented to participate. Of the 23 caregivers who consented and were available to complete the survey, 4 patient-caregiver dyads were subsequently excluded: 1 patient diagnosed with end-stage heart failure returned to their COO for what was thought to be their EOL, but their condition improved and they remained living in their COO at the time of data collection; and 3 patients died in hospice care in the U.S. Ultimately, 19 patient and caregiver dyads were included in the study (Figure 1).

Regarding the 3 patients who were excluded because they died in the U.S.: 1 caregiver stated that they were not aware that their loved one desired to return to their COO; another caregiver stated that they were informed by a physician that their loved one was too sick to travel and died in the U.S. under hospice care 6 weeks later; and the other caregiver stated that their loved one returned to their COO and visited with family but decided to return to the U.S. for their final days.

Demographic and clinical information for the repatriated deceased patients is shown in Table 1. The patients had a variety of diagnoses and co-morbidities. Most were male, married, Catholic, and had public insurance. Of the 19 caregivers, most (*n* = 12/19, 63.2%) were children of the deceased patients at EOL. The remaining caregivers identified as the deceased’s sibling (*n* = 2/19, 10.5%), partner (*n* = 1/19, 5.2%) or had another relationship to the deceased (*n* = 4/19, 21.1%, grandson, cousin, or declined to answer). Fourteen of the caregivers (*n* = 14/19, 73.7%) identified themselves as the primary caregiver. Of the 5 caregivers who did not identify as the primary caregiver, 3 (*n* = 3/5, 60%) were children of the deceased patient. Of these 3 caregivers, 2 identified other children as the primary caregiver(s) and 1 identified a sibling of the deceased as a primary caregiver. Almost all the deceased patients (*n* = 17/19, 89.5%) had additional people available to take care of them while living in the U.S., including additional family (*n* = 9/19, 52.9%), friends or neighbors (*n* = 5/19, 29.4%), or a paid home attendant (*n* = 3/19, 17.6%). Most of the deceased patients (*n* = 16/19, 82.4%) had lived in the U.S. for at least 1 year, with a range of 1–50 years and a median of 21.5 years. Three (*n* = 3/19, 15.8%) of the deceased patients had only lived in the U.S. for weeks to months. The interviews were conducted in Spanish with 10 of the caregivers, and in English for 9 of the caregivers.

**Table 1.** Demographic and clinical information for repatriated patients who died in their country of origin (*N* = 19) Table 1 long description.

Characteristic	*N* (%)
Sex	
Male	10 (52.6%)
Female	9 (47.4%)
Partnership status	
Single	6 (31.6%)
Married	9 (47.4%)
Widowed	1 (5.3%)
Unknown	3 (15.8%)
Insurance type	
Medicaid/medicare	15 (79.0%)
Private	3 (15.8%)
Self-pay	1 (5.3%)
Religion	
Catholic	13 (68.4%)
Not religious	1 (5.3%)
Jewish	1 (5.3%)
Unknown	4 (21.0%)
Diagnoses	
Gynecologic cancer	2 (10.5%)
Breast cancer	2 (10.5%)
Gastrointestinal cancer	3 (15.8%)
Hematologic cancer	1 (5.3%)
Other cancer	4 (21.0%)
Other non-cancer disease	7 (36.8%)
Comorbidities	
Cardiopulmonary	12 (63.1%)
Endocrine	6 (31.6%)
Psychiatric	3 (15.8%)
Neurologic	1 (5.3%)
Gastrointestinal	1 (5.3%)
Other	1 (5.3%)

Documentation in the U.S. electronic medical record provided insight into communication between patients and their U.S.-based clinical teams. In addition to documenting the patient’s desire to return to the COO for EOL care, clinician notes commonly reflected brief counseling regarding travel safety, and coordination with social work or case management. However, the depth and detail of these discussions were inconsistently documented.

### Travel back to country of origin

Based on caregiver survey responses, all patients wished to return to their COO at EOL. Most returned to the Dominican Republic (*n* = 15/19, 78.9%), 2 (10.5%) returned to Ecuador, 1 (5.3%) returned to Colombia, and 1 (5.3%) returned to Barbados. Six (31.6%) of the patients traveled to their COO directly from the hospital in the U.S., and the rest traveled from their home (*n* = 11, 57.9%), nursing home or rehabilitation facility (*n* = 1, 5.3%), or a hotel (*n* = 1, 5.3%). None of the patients experienced complications traveling to the airport in the U.S. Two of the patients had problems in transit from the airport in the U.S. to the airport in their country of origin: 1 patient had an episode of emesis on the plane, and the other patient missed their initial flight because the airline staff refused to let the patient on the plane until they spoke to medical staff, despite having a letter clearing her for travel from her oncologist. Two patients had difficulty traveling from the airport in their COO to their destination due to mobility issues that made it challenging to get into a vehicle for 1 patient, and to maneuver with a wheelchair in transportation that was not wheelchair friendly for the other patient.

### End-of-life care in COO

Most patients (*n* = 16/19, 84.2%) had a different primary caregiver in their COO than in the U.S.; and most patients (*n* = 15/19, 78.9%) had other people available to take care of them in their COO in addition to the primary caregiver, such as additional family (8/15, 53.3%), friends or neighbors (2/15, 13.3%), or a paid home attendant (5/15, 33.3%).

In the week leading up to the patients’ deaths, most patients (*n* = 13/19, 68.4%) did not go to the emergency room, hospital, or ICU. Of the 6 patients who did seek care at a medical facility, 1 went to the hospital and was subsequently admitted to the ICU, 4 were admitted to the hospital only (not admitted to the ICU), and 1 went to the emergency department but was not subsequently admitted to the hospital.

The caregivers were asked what interventions were received by the patient in their last week of life (Table 2): 7 received intravenous fluids (5 in the hospital, 2 at home in an outpatient setting), none received supplementary nutrition, none were placed on a ventilator, and 1 received alternative treatment. The alternative treatments described included herbs, oils, and massage. Two of the patients were seen by palliative care providers in an outpatient setting (i.e., not in the emergency department or hospital).

**Table 2.** End-of-life care and outcomes for repatriated patients who died in their country of origin (*N* = 19) Table 2 long description.

End-of-life care interventions	Yes, N (%)	No, N (%)	Unknown
Was the patient seen by a palliative care provider?	2 (10.5)	15 (78.9)	2 (10.5)
Did the patient receive any type of hydration other than by mouth, such as intravenous fluids?	6 (31.6)	11 (57.9)	2 (10.5)
Did the patient receive any type of nutrition other than by mouth, such as total parenteral nutrition or tube feeds?	0 (0.0)	18 (94.7)	1 (5.3)
Was the patient placed on a ventilator?	0 (0.0)	19 (100)	0 (0.0)
Did the patient receive any alternative treatment?	1 (5.3)	16 (84.2)	2 (10.5)
Was there a hope that the alternative treatment would provide a cure?	1 (5.3)	16 (84.2)	2 (10.5)
**Symptom burden**			
Did the patient have pain?	12 (63.2)	6 (31.6)	1 (5.3)
Was the pain controlled?	11 (91.7)	1 (16.7)	0 (0.0)
Did the patient have shortness of breath?	12 (63.2)	6 (31.6)	1 (5.3)
Was the shortness of breath controlled?	11 (91.7)	1 (8.3)	0 (0.0)
Did the patient have nausea and vomiting?	5 (26.3)	12 (63.2)	2 (10.5)
Was the nausea and vomiting controlled?	3 (60.0)	2 (16.7)	0 (0.0)
Did the patient have severe agitation?	3 (15.8)	13 (68.4)	3 (15.8)
Was the severe agitation controlled?	1 (33.3)	2 (15.4)	0 (0.0)
**Death outcomes**			
Do you believe the location of death (home, hospital, other) was where the patient wanted to die?	14 (73.7)	4 (21.1)	1 (5.3)
Do you believe that the patient had a peaceful death?	16 (84.2)	1 (5.3)	2 (10.5)
Do you believe that the patient died with dignity?	16 (84.2)	2 (10.5)	1 (5.3)
Was the patient surrounded by the people who they desired to be present at the time of their death?	14 (73.7)	4 (21.1)	1 (5.3)
Do you believe the patient was happy with the decision to return to their home country?	17 (89.5)	0 (0.0)	2 (10.5)
Do you think returning to their home country enhanced the patient’s quality of life at the end of life?	16 (84.2)	0 (0.0)	3 (15.8)

Symptoms experienced by the patients in the last week of life and whether the symptoms were controlled are shown in Table 2. Twelve (*n* = 12/19, 63.2%) patients had pain or shortness of breath, which were well-controlled in all but one patient. Five patients (*n* = 5/19, 26.3%) had nausea and vomiting, controlled in 3 out of 5 patients; and 3 (15.8%) had severe agitation, controlled in 1 out of 3 patients.

### Caregiver-reported death experience

Most patients (*n* = 12, 63.2%) lived for at least a month after they arrived in their COO (median 3 months, range 1–8 months), whereas 6 (31.6%) patients lived for less than a month after they arrived in their COO. Notably, 1 patient died 3 days after repatriation, 14 (73.7%) patients died at home, 4 (21.1%) died in hospital, and 1 (5.3%) died in transit to a medical facility. Of the 14 patients who died at home, all but 1 of the caregivers stated that was where the patient wanted to die; 1 caregiver was unsure. Of the 4 patients who died in the hospital, 1 caregiver stated that that was the patient’s preferred location of death, and 1 caregiver did not think that was the preferred location of death.

According to caregiver response, the majority of the patients died with peace and dignity (*n* = 16, 84.2%) at their preferred location, surrounded by the people who they desired to be present at the time of their death (*n* = 14, 73.7%) (Table 2). Most caregivers reported that their loved one was happy with the decision to return to their COO (*n* = 17, 89.5%), and that returning to their COO improved their QOL at EOL (*n* = 16, 84.2%). One patient did not have a peaceful death based on the caregiver survey response. This patient experienced bleeding and pain at home and died in transit to a medical facility. Most patients died surrounded by desired family and/or friends (“yes”: *n* = 14, 73.7%; “no”: *n* = 4; “unknown”: *n* = 1). Of the 4 patients for whom the caregiver reported that they were not accompanied by the desired people, 2 died at home. Of the 2 that died at home, 1 was alone (died unexpectedly overnight of a stroke), and the other had some family present, but not all the family they wished to be present arrived in time. The other 2 who were not accompanied by the desired people either died in transit to a medical facility or at the hospital.

Overall, QOD or the last week of life was rated as favorable by the caregivers, with an average score of 4.2 (range 1–5). The caregiver of the patient who died in pain and bleeding in transit to a medical facility gave a rating of 1. The caregivers of a patient who died alone in a hospital gave a rating of 2. However, the caregiver of this patient stated that the patient had been happy that she had returned to her COO at EOL.

## Discussion

Studies documenting the repatriation experiences of terminally ill patients and their subsequent QOD are essential for informing clinical practice and guiding institutional policies to support and facilitate this EOL care goal. In this study, we found that patients who returned to their COO generally encountered minimal challenges during travel and experienced a favorable QOD without high-intensity life-sustaining treatments. These findings suggest that repatriation at the EOL is both feasible and meaningful and should be considered during goals of care discussions for immigrant patients.

Most patients in our study returned to their COO with only minor problems during transit. A few experienced difficulties with boarding the plane or with local ground transportation upon arrival in their COO due to health-related limitations, but these issues were not insurmountable. This contrasts with case reports of undocumented or uninsured immigrants facing significant barriers to repatriation, including language challenges, limited social support, lack of insurance, and fears of deportation (Pan et al. 2004; Jaramillo and Hui 2016; Metchnikoff et al. 2018). These barriers often prevent access to treatment or may make travel infeasible, highlighting the complex interplay between legal status, health-care access, and EOL options. While we did not specifically assess documentation status, it is likely that most, if not all, patients in our cohort were documented, as the majority had either public or private insurance. Given the observational nature of our study, we cannot determine whether insurance or documentation status contributed to the generally positive repatriation experiences of our cohort compared to published case reports. This underscores the need for further research to identify factors that predict successful repatriation.

Caregivers in our study emphasized that their repatriated loved ones valued both the location of death and being near family, reinforcing the cultural importance of familism – family-centered care decision-making – among Latinx and immigrant populations (Smith et al. 2009). Prior research has shown that immigrant patients often experience disparities in EOL care, including higher rates of in-hospital death, contrary to their preferences (Tergas et al. 2022a). These disparities may stem from systemic barriers that prevent alignment of care with patient values. Our findings suggest that, for some immigrant patients, repatriation may offer a path to more culturally congruent EOL care; however, these insights also underscore the need for system-level changes within the U.S. to ensure that all patients, regardless of immigration status, can access care that aligns with their values and preferences.

Importantly, the patients in our cohort generally avoided aggressive medical interventions near death, which may have contributed to a favorable QOD. Most did not receive ICU-level care or invasive treatments, and symptom control was typically adequate. This aligns with previous evidence that favorable QOD includes symptom relief, avoidance of unnecessary hospitalizations, and death in the preferred location (Zhang et al. 2012; Alawneh and Anshasi 2021; Ortiz-Ortiz et al. 2021). In the U.S., racial, ethnic, and immigrant-related disparities in EOL care persist. Patients from non-White or immigrant backgrounds are more likely to receive intensive EOL care and less likely to die at home or in hospice settings (Stephens et al. 2020; Ferrario et al. 2021; Umaretiya et al. 2021). Our findings suggest that repatriation may have enabled some immigrant patients to avoid these disparities and experience a more peaceful, value-concordant end of life.

Data on the interplay between proximity to loved ones and aggressive medical interventions at EOL is limited. However, prior work has highlighted the importance of family involvement in EOL decision-making (Nakamura et al. 2010). The absence of a health-care proxy often results in health-care teams making decisions for this patient population.^13^ Given that hospital systems tend to default to high-intensity EOL care, this may lead to more aggressive EOL care against the patient’s true wishes (Dzeng et al. 2023). This is reflected in existing data on advanced-stage cancer patients who receive high-intensity EOL care within a short timespan from passing away (Hugar et al. 2021; Hicks-Courant et al. 2022). These practiced appear more frequent in minority-serving institutions (Wasp et al. 2020), underscoring the need for more culturally aligned care planning.

**Figure 2.**
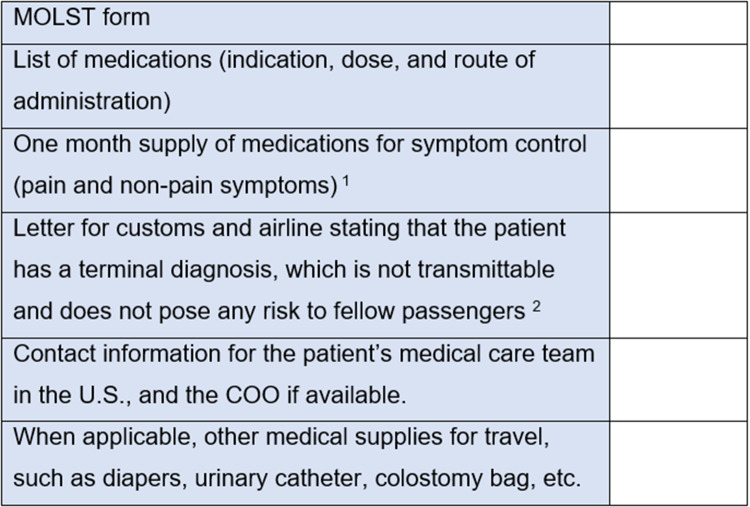
Checklist for patient travel preparation including medical forms, medication supply and contact information. Figure 2 long description.

Our findings underscore the importance of early, culturally sensitive goals-of-care conversations that include the option of repatriation. Because minoritized groups are more likely to die in hospitals against their preferences, facilitating alignment between values and care received is an important step toward equity (Stephens et al. 2020; Umaretiya et al. 2021). Early palliative care involvement and home-based palliative care services have been linked to increased rates of death at home (Kaye et al. 2018; Shabnam et al. 2022; Vestergaard et al. 2023). Furthermore, implementation of state-level legislation regarding palliative care delivery has been linked to higher rates of patients dying at home or in hospice (Quan Vega et al. 2023). We offer a practical checklist (Figure 2) to aid clinicians in supporting repatriation.

Our findings highlight the need for earlier and more structured communication regarding repatriation as a legitimate and culturally important EOL option, including routinely integrating repatriation into goals–of–care discussions using culturally informed approaches. Clinicians can more intentionally explore patients’ underlying hopes, values, and geographic preferences by asking questions that uncover what or who feels most important to them as their illness progresses. For example, “As we think about what’s most important to you in the time ahead, are there any places or people that feel especially important to you right now?” or, more explicitly for immigrants, “For many people living far from their home country, returning home is important to them. Is this something we should explore?” Because patients with serious illness often become too sick or medically unstable to travel once complications arise, these conversations are most effective when initiated early, ideally in the outpatient setting, using permission–seeking language such as, “I worry that despite this treatment, your cancer is continuing to progress. Would it be okay to talk about what could lie ahead?” Ensuring early involvement of social work, case management, and palliative care can help assess feasibility, mobilize resources, and coordinate logistics, while standardized documentation of these discussions can support continuity and reduce barriers to fulfilling this deeply value–based preference.

Beyond the practical checklist included in this study, additional interventions may further support successful repatriation and promote a high quality of death for immigrant patients. Examples include clinician educational interventions on culturally sensitive communication and the practical aspects of EOL travel, such as medical contraindications to flying, required documentation, and available resources in patients’ home countries. Institutional pathways or algorithms, along with partnerships with consulates and community organizations, may help streamline travel coordination. Establishing a strong, trust–based therapeutic alliance (Mack et al. 2009; Tergas et al. 2022b) may facilitate early goals–of–care conversations before patients become too medically fragile to travel. Future research should assess facilitators of and barriers to successful repatriation, and evaluate symptom management using validated patient–reported outcome tools, such as the Edmonton Symptom Assessment System (Hui and Bruera 2017). Larger studies are also needed to determine whether repatriation meaningfully improves QOD across diverse populations and destination countries.

This study has several limitations. This retrospective, single-institution cohort study was limited by a small sample size, partly due to relocation and isolation of patients and caregivers during the COVID-19 pandemic. These factors also limit the generalizability of our findings. Additionally, patient demographics may have contributed to low participation rates. An additional limitation is the possible discordance in EOL wishes of patients and their caregivers. Moreover, while our study emphasized objective measures of EOL care, certain aspects of QOD are inherently subjective and difficult to quantify (Curtis et al. 2013). This study also did not account for broader sociopolitical or economic factors that may influence repatriation preferences and processes, such as ongoing policy changes surrounding immigration.

In conclusion, our findings suggest that immigrant patients who wish to die in their COO and are able to do so may experience high-quality, culturally congruent EOL care. Repatriation should be discussed proactively during EOL goals-of-care planning. As immigration policies evolve, it is crucial that health-care systems respond to the needs of immigrant populations with compassion and flexibility. Future research should explore the key factors that facilitate successful repatriation and develop interventions to support this process.

## Data management and sharing

Data is subject to third-party restrictions.

## Figure 1 Long description

The flowchart illustrates the process and outcomes for patients desiring to return to their country of origin (COO). It starts with 53 patients expressing this desire. Caregivers (CG) were successfully contacted for 32 patients. Out of these, 27 caregivers enrolled. The chart shows that 21 caregivers were not contacted, 5 refused to participate and 4 enrolled but were later not able to be contacted. The outcomes are shown at the bottom: 19 patients died in COO, 3 died in the U.S. and 1 patient did not die.

Navigate back to Figure 1.

## Figure 2 Long description

A checklist detailing items necessary for patient travel preparation. It includes a MOLST form, a list of medications with indication, dose and route of administration and a one-month supply of medications for symptom control, covering both pain and non-pain symptoms. There is a letter for customs and airlines stating the patient's terminal diagnosis, which is not transmissible and poses no risk to fellow passengers. Contact information for the patient's medical care team in the U.S. and the COO is provided if available. Additionally, it mentions other medical supplies for travel, such as diapers, urinary catheter and colostomy bag, if applicable.

Navigate back to Figure 2.

## Table 1 Long description

The table provides demographic and clinical information for 19 repatriated patients who died in their country of origin. A majority of the patients were male (52.6%) and married (47.4%). Most had Medicaid/Medicare insurance (79%) and identified as Catholic (68.4%). The most common diagnosis was non-cancer diseases (36.8%), followed by various cancers, with gastrointestinal cancer being the most prevalent at 15.8%. Cardiopulmonary comorbidities were present in 63.1% of patients, indicating a significant health burden. The data highlights the predominance of public insurance and religious affiliation, with a notable presence of cardiopulmonary issues among the patients.

Navigate back to Table 1.

## Table 2 Long description

The table examines end-of-life care interventions, symptom burden, and death outcomes for 19 repatriated patients. Only 10.5% were seen by a palliative care provider, while 63.2% experienced pain and shortness of breath. Despite this, 84.2% of patients were believed to have died peacefully and with dignity. A significant 89.5% were thought to be happy with their decision to return to their home country, and 84.2% believed it enhanced their quality of life. The data suggests a gap in palliative care access but highlights positive perceptions of repatriation's impact on end-of-life experiences.

Navigate back to Table 2.
